# Supplementary material for: Serum Osmolarity and Vasopressin Concentration in Acute Heart Failure—Influence on Clinical Course and Outcome
Source: Biomedicines. 2022 Aug 20;10(8):2034. doi: 10.3390/biomedicines10082034 (PMC9405797; doi:10.3390/biomedicines10082034)
Supplement: Supplementary file 1 [file biomedicines-10-02034-s001.zip › biomedicines-1770598-supplementary.pdf]

Table S1. Univariate and multivariate analysis to establish factors influencing serum osmolarity.

Serum osmolarity				
Variable	Correlation		Multivariate model*	
	r	p	b-coefficient	p
Age, years	0.320	<0.001	0,262	<0.001
Systolic Blood Pressure, mmHg	0.140	0.010	-0.037	0.383
Diastolic Blood Pressure, mmHg	0.123	0.024	0.074	0.347
Body Weight, kg	0.023	0.706	-	-
Ejection fraction, %	0.058	0.322	-	-
Serum potassium concentration, mmol/l	0.200	<0.001	3.826	0.008
Hemoglobin, g/dl	-0.158	0.003	-0.702	0.134
Lactate, mmol/l	-0.119	0.039	0.315	0.695
CRP, mg/l	-0.074	0.179	-	-
NT-proBNP, pg/ml	0.149	0.006	0.0001	0.296
Albumin, g/dl	0.058	0.295	-	-
Bilirubin, mg/dl	-0.137	0.012	-0.657	0.325

*adjusted for: Age, Systolic Blood Pressure, Diastolic Blood Pressure, Serum potassium concentration, Hemoglobin, Lactate, NT-proBNP, Bilirubin

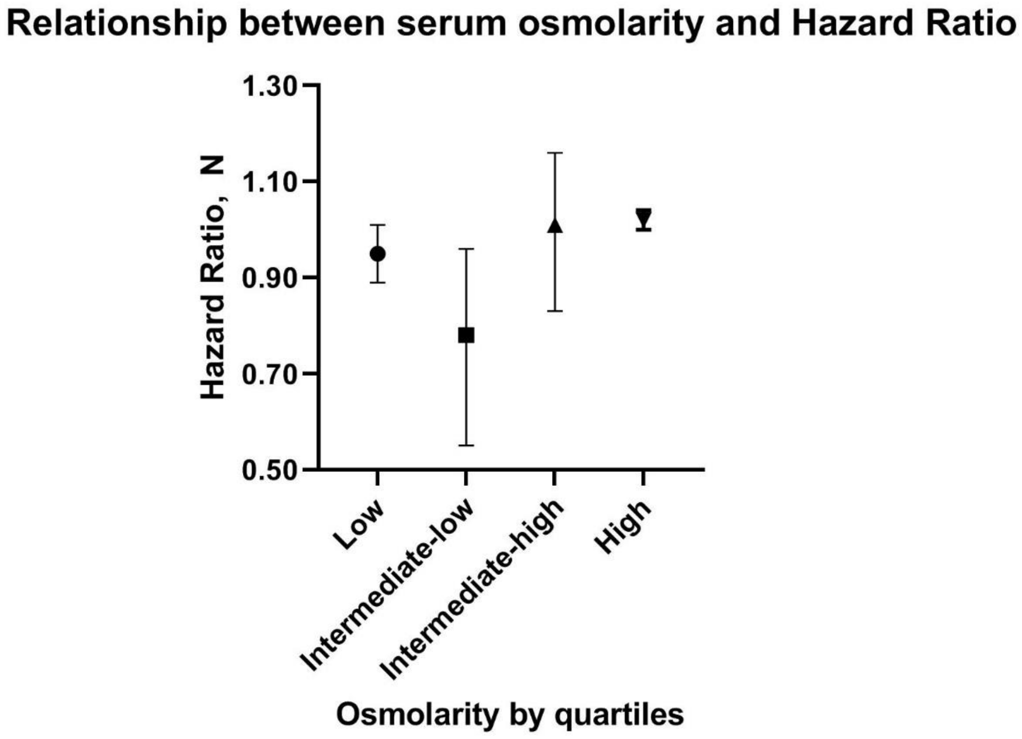

Figure S1. Relationship between serum osmolarity and Hazard Ratio

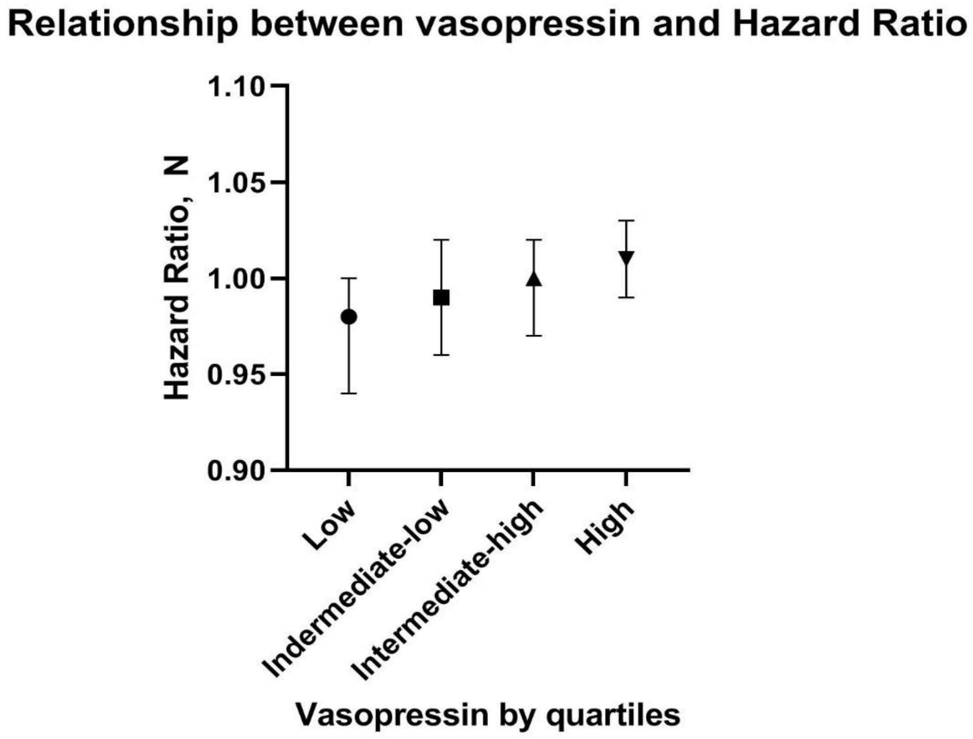

Figure S2. Relationship between vasopressin and Hazard Ratio
